# Aberrant dynamic functional network connectivity in type 2 diabetes mellitus individuals

**DOI:** 10.1007/s11571-022-09899-8

**Published:** 2022-11-21

**Authors:** Wenjiao Lyu, Ye Wu, Haoming Huang, Yuna Chen, Xin Tan, Yi Liang, Xiaomeng Ma, Yue Feng, Jinjian Wu, Shangyu Kang, Shijun Qiu, Pew-Thian Yap

**Affiliations:** 1 Department of Radiology, The First Affiliated Hospital of Guangzhou University of Chinese Medicine, Guangzhou, Guangdong China; 2 Department of Radiology and Biomedical Research Imaging Center (BRIC), University of North Carolina at Chapel Hill, Chapel Hill, NC USA; 3 Department of Endocrinology, The First Affiliated Hospital of Guangzhou University of Chinese Medicine, Guangzhou, Guangdong China; 4 Department of Radiology, Jingzhou First People’s Hospital of Hubei Province, Jingzhou, Hubei China; 5 School of Computer Science and Engineering, Nanjing University of Science and Technology, Nanjing, Jiangsu China

**Keywords:** Resting-state functional magnetic resonance, Type 2 diabetes mellitus, Cognitive impairment, Dynamic functional network connectivity, Intrinsic connectivity networks

## Abstract

An increasing number of recent brain imaging studies are dedicated to understanding the neuro mechanism of cognitive impairment in type 2 diabetes mellitus (T2DM) individuals. In contrast to efforts to date that are limited to static functional connectivity, here we investigate abnormal connectivity in T2DM individuals by characterizing the time-varying properties of brain functional networks. Using group independent component analysis (GICA), sliding-window analysis, and k-means clustering, we extracted thirty-one intrinsic connectivity networks (ICNs) and estimated four recurring brain states. We observed significant group differences in fraction time (FT) and mean dwell time (MDT), and significant negative correlation between the Montreal Cognitive Assessment (MoCA) scores and FT/MDT. We found that in the T2DM group the inter- and intra-network connectivity decreases and increases respectively for the default mode network (DMN) and task-positive network (TPN). We also found alteration in the precuneus network (PCUN) and enhanced connectivity between the salience network (SN) and the TPN. Our study provides evidence of alterations of large-scale resting networks in T2DM individuals and shed light on the fundamental mechanisms of neurocognitive deficits in T2DM.

## Introduction

There are approximately 536.6 million adults with diabetes mellitus worldwide and this number may exceed 783.2 million by 2045, 90–95% of which are type 2 diabetes mellitus (T2DM) (Sun et al. 2022). Epidemiological studies have established that individuals with T2DM have a significantly higher risk of incident cognitive impairment (Biessels et al. 2014; Rawlings et al. 2019). Among the medical complications of T2DM, cognitive impairment is regarded as a major public health problem (Stoeckel et al. 2016) lacking in effective therapy and associated with huge financial burden. Despite numerous brain imaging studies focused on T2DM patients over the past 20 years (Biessels and Reijmer, 2014; Moran et al. 2017; Biessels and Despa, 2018), the underlying neuromechanism of cognitive deficits in T2DM patients remains elusive.

As previous studies indicated, changed cerebral blood flow, altered hemodynamic response function and impaired neurovascular coupling have been find in T2DM patients (Duarte et al. 2015; Hu et al. 2019; Ryan et al. 2014). Resting-state functional MRI (rs-fMRI) quantifies brain activation depending on variations in blood oxygenation in response to neural activities and may provide information on abnormal functional connectivity in T2DM (Xia et al. 2015; Cui et al. 2016; Zhang et al. 2021; Huang et al. 2020a). However, most studies to date are based on functional connectivity confined to regions of interest (ROIs) rather than the whole brain. Furthermore, these studies neglect the fact that functional connectivity of the brain is not static over time and, on the contrary, fluctuates constantly (Allen et al. 2014; Chang and Glover, 2010; Hutchison et al. 2013; Preti et al. 2017; Calhoun et al. 2014; Alonso Martinez et al. 2020; Mennigen et al. 2019).

Functional network connectivity (FNC) was proposed to evaluate the connectivity between functional networks (Jafri et al. 2008). Dynamic functional network connectivity (dFNC) information can be captured using a sliding-window method (Zalesky et al. 2014; Allen et al. 2014). Some studies suggested that alterations of dFNC may be related to some neurophysiological processes and neuropsychiatric diseases (Wang et al. 2020; Chen et al. 2018; Allen et al. 2014; Calhoun et al. 2014; Zhi et al. 2018) and revealed that the functional connectivity between different brain regions as well as its dynamic reconfiguration were both crucial to the cognitive dysfunction. (Calhoun et al. 2014; Damaraju et al. 2014; Zhi et al. 2018; Sendi et al. 2020; Shine et al. 2016; Vatansever et al. 2015). Driven by the importance of dFNC analysis in the investigation of cognitive impairment, we speculate that this method will provide new clues in understanding the mechanism of cognitive impairment in T2DM. However, to the best of our knowledge, no published study to date has reported the dFNC characteristics across large-scale whole-brain intrinsic connectivity networks (ICNs) in T2DM individuals.

The main purpose of our study is to investigate abnormal dynamic information of brain activity in T2DM individuals. We employed group independent component analysis (GICA) (Fox et al. 2005; Mennigen et al. 2019) to extract and classify intrinsic connectivity networks (ICNs) (Mennigen et al. 2019; Calhoun et al. 2001; McKeown et al. 1998) from whole-brain rs-fMRI data, applied sliding windows and k-means clustering for dFNC analysis, and finally characterized inter-group differences in brain states generated by the dFNC procedure. We evaluated the temporal properties of each dFNC state, including the fraction time (FT), mean dwell time (MDT), and number of transitions (NT), as well as their correlations to MoCA scores. This study provides novel insights into the fundamental mechanisms of neurocognitive deficits related to T2DM.

## Methods

### Participants

The participants, who were between the ages of 18 and 65, right-handed, and should have no history of neuropsychiatric, cerebrovascular, tumor, autoimmune and any other diseases that might affect brain structure and cognitive function, as well as without MRI contraindications, were recruited from December 2019 to December 2020. The diagnosis of T2DM was determined based on the criteria set by the American Diabetes Association (ADA) based on fasting plasma glucose (FPG) levels (≥ 7.0 mmol/L) or 2-h oral glucose tolerance test (OGTT) glucose levels (≥ 11.1 mmol/L). After image preprocessing (see section “Data Preprocessing”), two of the 102 individuals were eliminated, resulting in a final number of 100 participants ultimately involved in fMRI data analysis (54 T2DM individuals and 46 healthy controls). The study was authorized by the Medical Research Ethics Committee of Guangzhou University of Chinese Medicine (NO. K2019-143). All participants were fully informed of the purpose, process, and risks of the study before signing the informed consent.

### Cognitive assessment

Before image acquisition, the Chinese version of the Montreal Cognitive Assessment (MoCA) scale testing was administered to assess the general cognitive function of each participant (Nasreddine et al. 2005). MoCA involves a 30-point scale with 7 modules, corresponding to visual spatial executive function, naming ability, memory, attention, language function, abstraction, and orientation. If the number of years of education is not more than 12 years, one point is added, provided that the final score does not exceed 30 points. A score of less than 26 indicates cognitive impairment.

### Image acquisition

A 3 T Siemens MAGNETOM Prisma MRI scanner (Siemens Healthcare, Erlangen, Germany) with a 64-channel head coil was used for MRI acquisition in the First Affiliated Hospital of Guangzhou University of Chinese Medicine (Guangzhou, Guangdong, China). The 3D T1-weighted MPRAGE sequence was employed with the following parameters: TE = 2.98 ms, TR = 2530 ms, Flip angle = 7^◦^, FOV = 256 × 256 mm^2^, slice thickness = 1.0 mm, voxel size = 1.0 × 1.0 × 1.0 mm^3^, TA = 5 min 58 s.

For rs-fMRI, simultaneous multi-slice (SMS) imaging was employed with echo-planar imaging (EPI) sequence with the following parameters: TE = 30 ms, TR = 500 ms, flip angle = 60^◦^, FOV = 224 × 224 mm^2^, slice thickness = 3.5 mm, voxel size = 3.5 mm × 3.5 mm × 3.5 mm. For each participant, a total of 960 volumes (35 slices per volume) were collected in 8 min 7 s. To minimize head motion and scanner noise, foam pads and earplugs were used for all the participants. The participants were also instructed to keep their eyes closed, move as minimally as possible, relax, and let their minds wander freely without falling asleep.

### Data processing

The data was processed via SPM 12 (http://www.fil.ion.ucl.ac.uk/spm), GRETNA (version 2.0, http://www.nitrc.org/projects/gretna) (Wang et al. 2015) and MATLAB 2013b (The Mathworks Inc., Natick, MA, United States). The first 10 time points of each participant’s rs-fMRI data were discarded to eliminate the destabilizing effects that may be caused the maladjustment of the machine and participants at the beginning of the scan, leaving 950 images for subsequent analysis. Slice-timing correction was not conducted considering the TR = 500 ms, since prior investigation suggested that slice-timing is not necessary for short TR (Smith et al. 2013). Further pre-processing steps were as follows: registration of T1-weighted images to mean volume, spatial normalization of functional images to the standard Montreal Neurological Institute (MNI) space with warping parameters estimated from co-registered T1 images using Diffeomorphic Anatomical Registration Through Exponentiated Lie algebra (DARTEL) (Goto et al. 2013), reslicing the voxel size to 3 × 3 × 3 mm^3^, and smoothing of the normalized data with a 6 mm full width half max (FWHM) Gaussian kernel to increase signal-to-noise ratio. After data preprocessing, two of the 102 participants were excluded because of excessive head motion (displacement > 2 mm or rotation > 2^◦^). All steps above were repeated for the MRI data from the remaining 100 subjects because DARTEL normalization involves registration of all subjects (Ashburner, 2007).

### Independent component analysis

Spatial independent components (ICs) and their corresponding time courses were calculated using GICA implemented in the Group ICA of fMRI Toolbox (GIFT, version 4.0b, https://trendscenter.org/software/gift/) (Calhoun and Adali 2012; Calhoun et al. 2001; Lewis et al. 2020). According to previous studies (Allen et al. 2014; Rabany et al. 2019; Espinoza et al. 2019; Jiang et al. 2020; Mennigen et al. 2019; Huang et al. 2020b), the rs-fMRI data were decomposed by subject-level principal component analysis (PCA) for dimension reduction to 60 components. The data of the individuals were then concatenated temporally and reduced by group-level PCA to yield 40 independent components. The infomax algorithm from ICASSO was applied to derive the independent spatial map and time course of each component. The algorithm was repeated 20 times to improve the stability of the decomposition (Ma et al. 2011). The subject-specific spatial maps and time courses were back-reconstructed from group-level independent components given by GICA (Calhoun et al. 2001; Erhardt et al. 2011).

31 of the 40 obtained ICs were identified as ICNs and categorized into 12 networks (Shirer et al. 2012; Franco et al. 2009) based on the following criteria: (1) the peak cluster locations should be in gray matter, (2) the spatial distributions overlapped minimally with ventricular and edge regions of the brain, and (3) the time courses should be predominantly low-frequency signals (Allen et al. 2011).

### Dynamic functional network connectivity

Analysis was conducted using the temporal dFNC toolbox in GIFT (Allen et al. 2014; Mennigen et al. 2019; Jiang et al. 2020). The time courses of the 31 ICNs were linearly detrended, despiked, and band-pass filtered (0.01–0.15 Hz). The size of the sliding window used to calculate dFNC was set to 44 s (88TRs) according to previous studies, which show that dynamic information can be captured with a window length of 30–60 s (Hutchison et al. 2013; Shirer et al. 2012; Allen et al. 2014; Faghiri et al. 2018; Mennigen et al. 2019; Jiang et al. 2020). With a step size of 1 TR (= 0.5 s), the window was slid. By convolution a rectangular window with a Gaussian function (σ = 3), a tapered window was generated. A total of 862 dFNC matrices were yielded by calculating a full correlation matrix for each window across 31 ICNs.

K-means clustering was employed on the FNC matrices of all sliding windows of all subjects to detect recurring FNC patterns. The squared Euclidean distance algorithm (500 iterations and 150 replications) was applied to evaluate the similarity of functional connectivity patterns between various time windows (Malhi et al. 2019). Four whole-brain FNC recurring patterns were identified using the elbow method (ratio of within- to between-cluster distances) (Allen et al. 2014). Windows from all participants were clustered into four states by adopting cluster centroids as initializations. The following dFNC indices were computed (Mennigen et al. 2019; Jiang et al. 2020; Wang et al. 2020): (1) the fraction time (FT), which indicates the percentage of total time in a brain state, (2) the mean dwell time (MDT), which represents the time spent in a given state before shifting to a different, and (3) the number of transitions (NT) between discrete dynamic states.

### Statistical analysis

Two-sample *t*-tests were used to detect group differences in normally distributed data, and nonparametric Mann–Whitney *U*-tests were used for non-normally distributed data. The Chi-square test was used for categorical variables. The statistical differences between groups were controlled False Discovery Rate (FDR) correction. The relationships between MoCA scores and dynamic temporal properties were evaluated using Spearman partial correlation analysis controlled with age and education. *P* < 0.05 was regarded as statistically significant. All statistical analyses were carried out using R (version 4.1.0) on R-Studio (https://www.rstudio.com/).

### Validation analysis

To verify robustness, we repeated the dFNC analysis by fixing the window length (44 s/88 TRs) and varying the number of clusters (5 and 6) and fixing the number of clusters (4) and varying the window length (30 s/60 TRs and 60 s/120 TRs) (Wang et al. 2020). Detailed information can be found in supplementary materials.

## Results

### Demographics and clinical characteristics

There were no significant differences in terms of age, gender, years of education, systolic blood pressure (Systolic BP), and diastolic blood pressure (Diastolic BP) between the T2DM and HC groups. The HC group showed significantly higher MoCA scores compared with the T2DM group. Detailed demographic and clinical information for the T2DM and HC groups are displayed in Table 1.

**Table 1:** Demographics and Clinical Characteristics of the participants

	T2DM (N = 54)	HC (N = 46)	*t* /*U* /*χ2*	*P -*Value
Gender			3.52	0.0607
Female	24 (44.4%)	30 (65.2%)		
Male	30 (55.6%)	16 (34.8%)		
Age (years)	46.0 (40.0, 54.0)	49.5 (35.5, 55.0)	1225.00	0.9091
Education (years)	12.0 (9.0, 14.0)	12.0 (9.0, 12.0)	1239.00	0.9859
Systolic BP (mmHg)	130 ± 17.4	125 ± 17.7	1.64	0.1046
Diastolic BP (mmHg)	85.2 ± 9.65	82.0 ± 8.86	1.74	0.0867
MoCA	27.0 (25.0, 28.0)	28.0 (27.0, 29.0)	838.50	0.0047^**^
T2DM Duration (years)	3.00 (2.00, 7.00)	NA	NA	NA
HbA1c (%)	8.75 (7.10, 11.08)	NA	NA	NA
FBG (mmol/L)	8.05 (7.05, 10.42)	NA	NA	NA
FINS (µIU/mL)	8.59 (5.30, 13.35)	NA	NA	NA

Data are presented as N (%), median (Q1, Q3) and mean ± SD. T2DM, Type 2 diabetes mellitus group; HC, healthy control group; Systolic BP, systolic blood pressure; Diastolic BP, diastolic blood pressure; MoCA, Montreal cognitive assessment; HbA1c, Hemoglobin A1c; FBG, fasting blood glucose; FINS, fasting insulin. Chi-square test was used for statistical difference of gender. Two sample *t*-test was used for statistical group differences of systolic blood pressure and diastolic blood pressure. Nonparametric Mann–Whitney *U* test was performed for group comparison of the remaining variables. Two asterisks (**) indicate the significant level with *P* < 0.01

### Identification of functional networks

**Fig.1:**
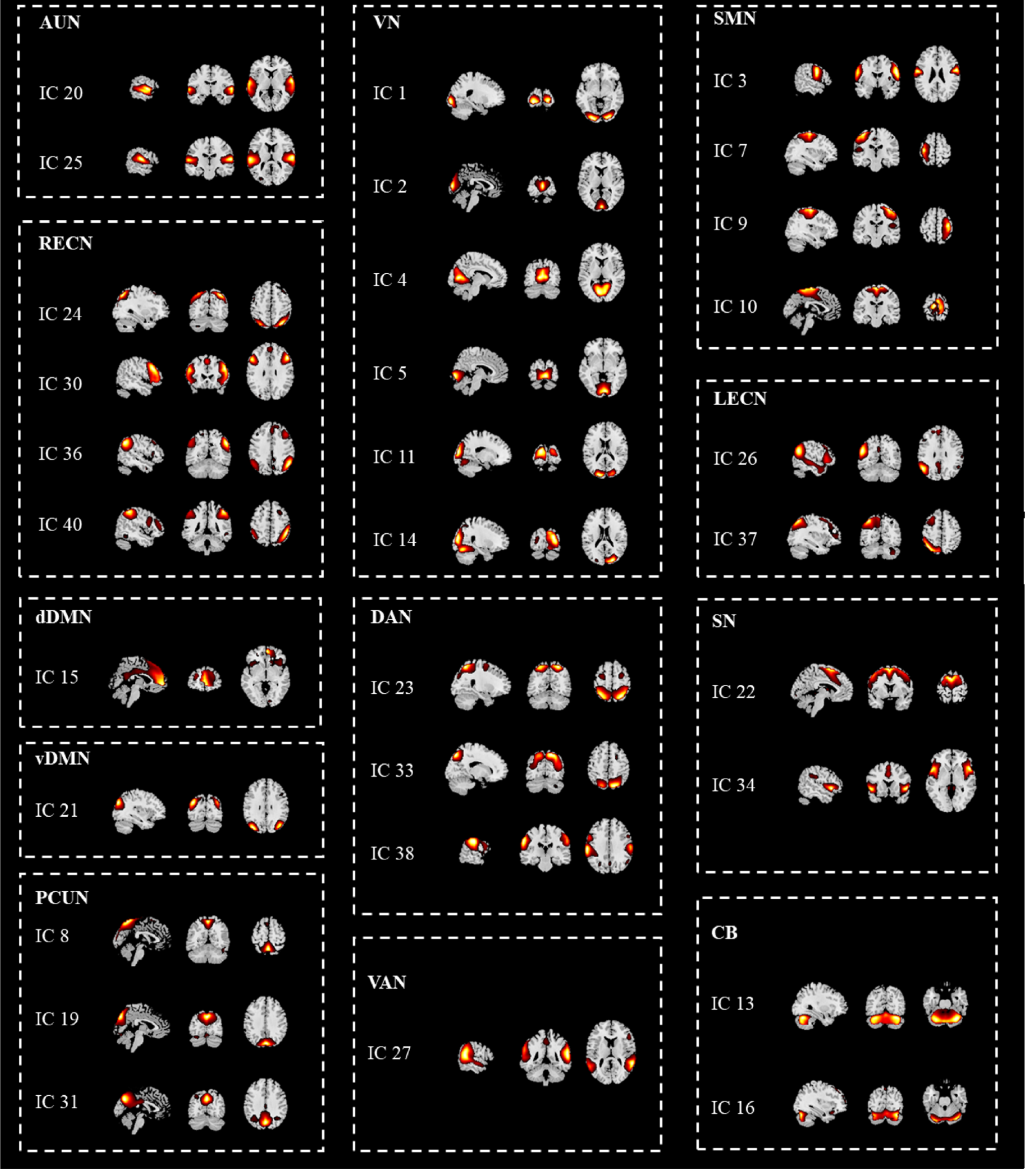
Spatial maps of the 31 intrinsic connectivity networks (ICNs). The 31 ICNs were grouped into 12 functional networks based on their anatomical and functional properties. AUN, auditory network; SMN, sensorimotor network; VN, visual network; LECN, left executive control network; RECN, right executive control network; dDMN, dorsal default mode network; vDMN, ventral default mode network; PCUN, precuneus network; SN, salience network; DAN, dorsal attention network; VAN, ventral attention network; CB, cerebellar network. dDMN, vDMN and PCUN belong to default mode network. The spatial maps of each domain are overlaid onto a standard template and represented by color. (Color figure online)

Twelve functional networks were ultimately summarized from the 31 ICs extracted via ICA (Fig. 1), including the auditory network (AUN: IC 20 and IC 25), visual network (VN: IC 1, IC 2, IC 4, IC 5, IC 11 and IC 14), sensorimotor network (SMN: IC 3, IC 7, IC 9 and IC 10), left executive control network (LECN: IC 26 and IC 37), right executive control network (RECN: IC 24, IC 30, IC 36 and IC 40), dorsal default mode network (dDMN: IC 15), ventral default mode network (vDMN: IC 21), precuneus network (PCUN: IC 8, IC 19 and IC 31), salience network (SN: IC 22 and IC 34), dorsal attention network (DAN: IC 23, IC 33 and IC 38), ventral attention network (VAN: IC 27) and cerebellar network (CB: IC 13 and IC 16). The dDMN, vDMN and PCUN are the three subnetworks of the DMN.

### Dynamic functional network connectivity analysis

**Fig. 2:**
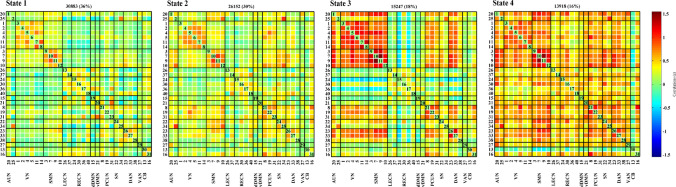
Four identified dFNC states using k-means clustering method derived from ICA components. Each cluster represents a particular dFNC state. The total number and percentage of the recurrence of each state are listed above each cluster. The colors in the matrices reflect positively correlations (green to red range) or negatively correlations (green to blue range). (Color figure online)

**Fig. 3:**
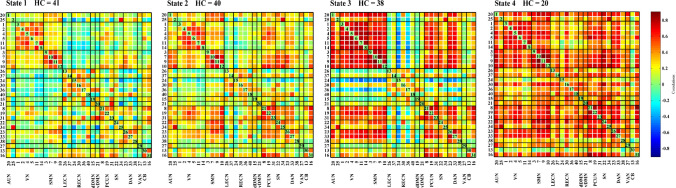
The dFNC matrices states of HC group. HC, healthy control group. The total number of HC participants involved in each state are listed above. The colors in the matrices reflect positively correlations (green to red range) or negatively correlations (green to blue range). (Color figure online)

**Fig. 4:**
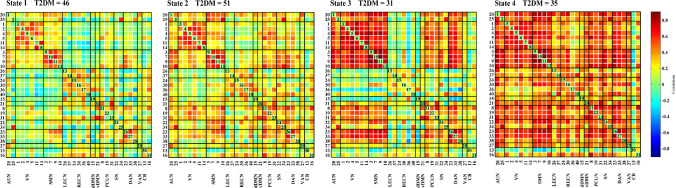
The dFNC matrices states of T2DM group. T2DM, Type 2 diabetes mellitus group. The total number of T2DM participants involved in each state are listed above. The colors in the matrices reflect positively correlations (green to red range) or negatively correlations (green to blue range). (Color figure online)

We used a sliding-window method to extract 862 windows from the time courses of all subjects and applied k-means clustering to group the dFNC matrices associated with these windows into four states, which respectively accounted for 36%, 30%, 18% and 16% of the windows. The centroids of the four states are shown in Fig. 2. State 1, the most common state, exhibited extensively weak inter-network and intra-network connectivity, but relatively high positive local intra-network connectivity within primary sensory networks (VN, SMN). State 2 was a hypoconnected state with weak inter-network and intra-network connectivity except slightly positive connectivity within the VN and SMN and between the SMN and SN. State 3 demonstrated strong positive inter-network and intra-network connectivity within the primary sensory networks (AUN, SMN, VN), as well as positive connectivity between these primary sensory networks and the DAN, but slightly negative inter-network and intra-network connectivity within the LECN, RECN, and dDMN. State 4 exhibited positive inter-network and intra-network connectivity involving almost all the networks, except for the slight negative connectivity between IC13 in CB domain and other ICNs. The centroids of the brain states of the HC and T2DM groups are shown in Figs. 3 and 4 respectively. Few subjects in the HC group experienced state 4 and few subjects in the T2DM group experienced state 3 (*P* < 0.05) (Table 2).

**Table 2:** The number of participants and temporal properties of two groups in each dFNC state

	T2DM (N = 54)	HC (N = 46)	*t* /*U* /*χ2*	*P -*value
NP-State1	46 (85.19%)	41 (89.13%)	0.08	0.7746
NP-State2	51 (94.44%)	40 (86.96%)	0.91	0.3403
NP-State3	31 (57.41%)	38 (82.61%)	6.24	0.0125^*^
NP-State4	35 (64.81%)	20 (43.48%)	3.75	0.0529
FT-State1	0.31 (0.04, 0.76)	0.17 (0.08, 0.60)	1309.50	0.6424
FT-State2	0.19 (0.07, 0.42)	0.18 (0.07, 0.61)	1202.00	0.7847
FT-State3	0.02 (0.00, 0.11)	0.20 (0.07, 0.41)	634.00	< 0.001^**^
FT-State4	0.12 (0.00, 0.42)	0.00 (0.00, 0.09)	1712.50	< 0.001^**^
MDT-State1	68.50 (23.88, 227.54)	54.60 (28.00, 111.25)	1355.50	0.4336
MDT-State2	47.00 (19.25, 76.30)	62.00 (20.75, 96.33)	1156.00	0.5543
MDT-State3	9.25 (0.00, 46.62)	72.80 (35.25, 107.75)	617.50	< 0.001^**^
MDT-State4	50.60 (0.00, 90.96)	0.00 (0.00, 50.12)	1687.50	< 0.01^**^
NT	8.39 ± 4.66	8.57 ± 4.31	− 0.20	0.8457

Data are presented as N (%), median (Q1, Q3) and mean ± SD. T2DM, Type 2 diabetes mellitus group; HC, healthy control group; NP, number of participants; FT, fraction time; MDT, mean dwell time; NT, number of transitions; Q1, the first quartile; Q3, the third quartile. Chi-square test was used for statistical difference of number of participants. Two sample *t*-test was used for statistical group differences of number of transitions. Nonparametric Mann–Whitney *U* test was performed for group comparison of the remaining variables. One asterisk (*) indicates significance level *P* < 0.05; Two asterisks (**) indicate significance level *P* < 0.01

**Fig. 5:**
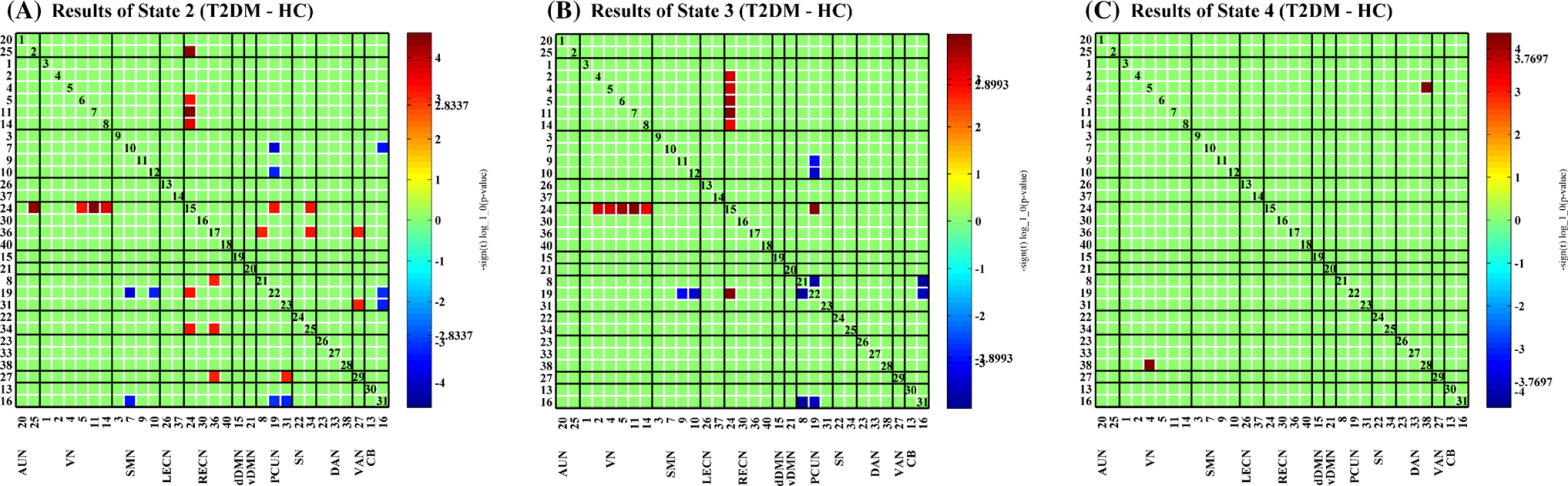
Significant group differences of dFNC in each state. T2DM, Type 2 diabetes mellitus group; HC, healthy control group. A, B and C illustrates significant group differences of dFNC between type 2 diabetes mellitus and healthy control group (T2DM − HC) in state 2, 3 and 4 respectively (two sample *t*-test, *P* < 0.05, FDR corrected). The results are displayed as -sign (t-statistic) × log10 (p-value). No meaningful value survived state 1. The color reflects higher connectivity (green to red range) or lower connectivity (green to blue range) in type 2 diabetes mellitus than healthy control group. (Color figure online)

The significant differences in connectivity strength between HC and T2DM individuals in dFNC states are shown in Fig. 5 (two-sample *t*-test, *P* < 0.05, pFDR = 0.05). We found that in state 2, the connectivity strength between the RECN and AUN/ VN/ PCUN/ SN/VAN, and between PCUN and VAN was significantly higher, but the connectivity strength between PCUN and SMN and CB was significantly lower in the T2DM group (Fig. 5a). Although significant group differences that survived the test were not as many as in state 2, state 3 showed a similar pattern: higher connectivity between RECN and VN/ PCUN, while prominent lower connectivity between PCUN and SMN/ CB in the T2DM group (Fig. 5b). State 4 displayed higher connectivity between the VN and DAN in the T2DM group than the HC group (Fig. 5c). No meaningful value survived in state 1.

### Temporal properties of dynamic functional network connectivity states

**Fig. 6:**
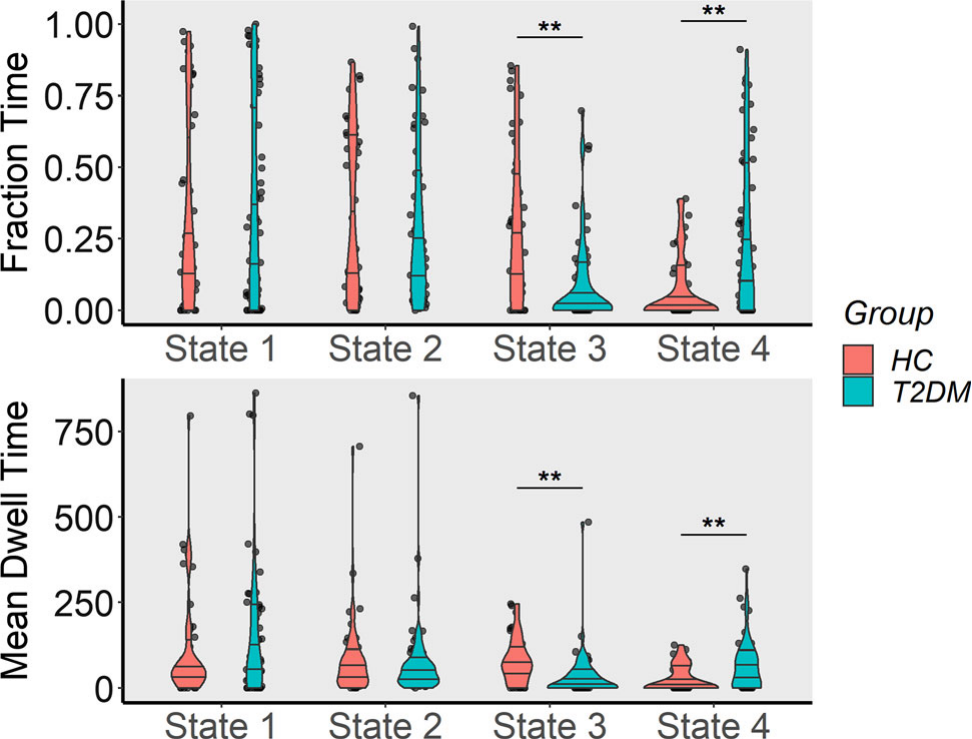
The comparisons between type 2 diabetes mellitus and healthy control group in fraction time and mean dwell time in each dFNC state. T2DM, Type 2 diabetes mellitus group; HC, healthy control group. The top violin figure is a comparison of the fraction time for each state of the two groups, the bottom violin figure is a comparison of the mean dwell time for each state of the two groups. Two asterisks (**) indicate significance level *P* < 0.01 with Mann–Whitney *U* test. (Color figure online)

Compared to the HC group, the T2DM group exhibited significantly decreased FT and MDT in state 3 (Mann–Whitney *U* = 634 and 617.5, respectively, *P* < 0.001, pFDR = 0.05) and remarkably increased FT and MDT in state 4 (Mann–Whitney *U* = 1712.5 and 1687.5, respectively, *P* < 0.01, pFDR = 0.05). The FT and MDT in state 1 and state 2 showed no statistically significant group differences (Fig. 6), as well as the NT. Detailed information on the temporal properties is shown in Table 2.

### Relationships between temporal properties and MoCA scores

**Fig. 7:**
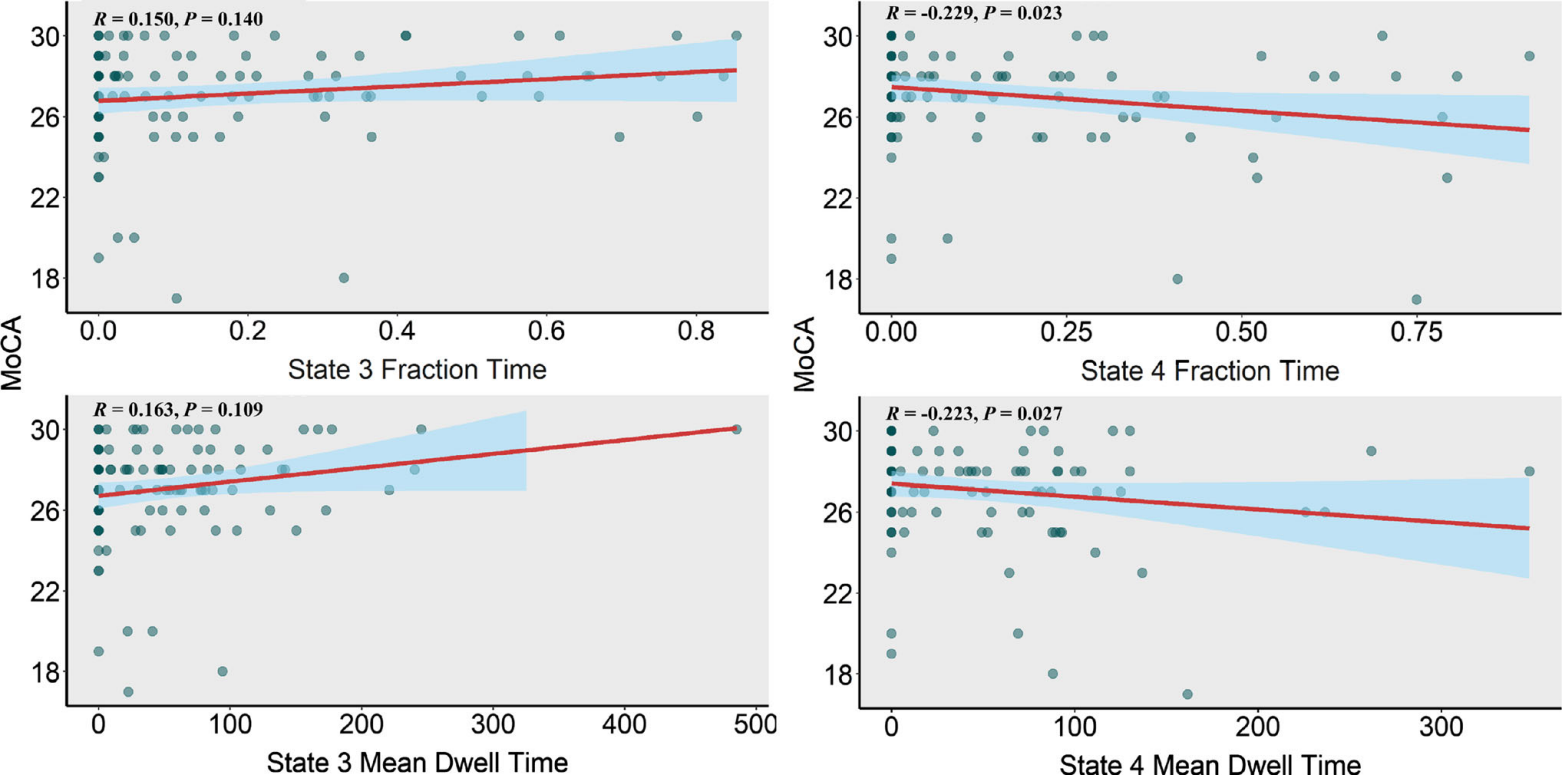
Correlations between MoCA scores and temporal properties of dFNC. With age and education as covariates, we found significant negative correlation between MoCA scores and the fraction time (Spearman correlation, *R* =  0.229, *P* = 0.023) as well as between MoCA scores and the mean dwell time (Spearman correlation, *R* =  0.223, *P* = 0.027) in state 4. We also found slightly positive correlations between MoCA scores and the fraction time as well as between MoCA scores and the mean dwell time in state 3, but not statistically significant (*P* > 0.05). No meaningful correlation survived between MoCA scores and temporal properties of dFNC in state 1 and state 2 (*R* was close to 0). (Color figure online)

We found significant negative correlations between MoCA scores and FT (*R* = − 0.229, *P* = 0.023) as well as between MoCA scores and MDT (*R* = − 0.223, *P* = 0.027) in state 4. Slight positive correlations, but not statistically significant, can be found between MoCA scores and FT (*R* = 0.150) as well as between MoCA scores and MDT (*R* = 0.163) in state 3 (Fig. 7). No significant correlation can be found between MoCA scores and other temporal properties in state 1 and state 2.

## Discussion

In the current study, we investigated aberrant dynamic connectivity in T2DM individuals. With GICA, we identified 31 ICN-related components and associate them with 12 brain networks. We estimated four recurring states via a sliding window method together with k-means clustering from the fMRI time courses. We analyzed the characteristics of each state and revealed significant differences between T2DM and HC groups in FT and MDT of state 3 and state 4. In addition, there was a significant negative correlation between the MoCA scores and FT/ MDT in state 4. In the T2DM group, the inter-network and intra-network connectivity decreased and increased respectively for the DMN and TPN. Our results also indicated enhanced connectivity between the SN and TPN in the T2DM group. Our findings shed light on the underlying neural basis of cognitive decline in T2DM individuals.

### Recurring brain states

Accordance with previous findings (Allen et al. 2014; Viviano et al. 2017; Gu et al. 2020), the most frequent recurring state, i.e., state 1, is a ‘sparsely connected state’ (Wang et al. 2020) with weak and diffused connectivity. This state is associated with self-referential thinking and sleepiness (Allen et al. 2018), is considered a steadier state with increased interoceptive awareness and decreased vigilance (Wang et al. 2020; Fu et al. 2019), and is regarded as representing the baseline connectivity pattern (Viviano et al. 2017). The frequent occurrence of this condition may imply that, in order to conserve energy, the human brain prefers to remain in a state with low information transmission (Gu et al. 2020). Although previous research showed that patients with brain disorders may stay longer in this state (Wang et al. 2020), our study indicated no inter-group differences in state 1 between T2DM and HC groups, suggesting that this state is stable and unaltered in T2DM.

State 2 was also associated with relatively weak and diffused connectivity. State 3 was a ‘highly segregated state’ (Wang et al. 2020) with greater modularity (Bonkhoff et al. 2020). Growing evidence suggests that Alzheimer’s disease (AD) (Brier et al. 2014), schizophrenia (Yang et al. 2016a) and stroke (Siegel et al. 2018) are potentially associated with abnormal modular properties. Modularity is also associated with cognitive function (Gallen et al. 2016; Wang et al. 2020) and learning capacity (Bassett et al. 2011). Our results showed that the FT and MDT of the T2DM group decrease remarkably in state 3. State 4 was least frequently recurring but was most connected. Strong connectivity was thought to be associated with cognitive function compensation (Voets et al. 2009; Yang et al. 2018). It is however also believed to be linked with neuropsychiatric dysfunction (Centeno and Carmichael, 2014; Liang et al. 2021; Tan et al. 2021). Our results showed that the FT and MDT of the T2DM group increased considerably in state 4. Both the FT and MDT in this state were negatively correlated with MoCA scores.

### Group differences

From the group differences in states 2, 3, and 4, we can observe in the T2DM group that (1) the connectivity strength between the DMN and the SMN/ CB, as well as within the DMN was reduced; and (2) the connectivity strength between the RECN and the AUN/ VN/ PCUN/ SN/ VAN, and between the DAN and the VN was increased. The RECN, DAN and VAN all belong to the TPN. The DMN, TPN and SN have crucial impact on cognitive status (Kim and Kim, 2021; Hasenkamp et al. 2012).

The identification of DMN is considered as one of the most prominent findings in cognitive neuroscience (Jenkins, 2019). This network was originally regarded as the task negative network (TNN) for its deactivation when the brain is occupied by a task (Fox et al. 2006). In comparison to other brain regions, the DMN is characterized by higher activity in the resting-state, which hence constitutes essential conscious experience (Smallwood and Schooler, 2006). Subsequent studies illustrate that the DMN is closely associated with sustaining the balance and steadiness of internal states during rest, mind wandering, internal recognition (such as autobiographical memory, perspective memory, self-reflection and first-person perspective) and social cognition (Buckner et al. 2008, Poerio et al. 2017, Raichle, 2015, Andrews-Hanna et al. 2014, Jenkins, 2019). Investigators recently suggest that the DMN is crucial for integrating external and internal information, as well as aligning thoughts and actions (Yeshurun et al. 2021). It has been well documented that reduced activity in DMN is related to cognitive damage (Jenkins, 2019; Bonnelle et al. 2011). Consistent with previous T2DM studies (Yang et al. 2016b; Cui et al. 2015; Musen et al. 2012; Tan et al. 2019; Chen et al. 2015), we detected impaired functional inter-network and intra-network connectivity of DMN in T2DM patients. Due to the pivotal role DMN played in cognitive function, we divided it into the dDMN, vDMN and PCUN (Shirer et al. 2012) to investigate connectivity alteration in each subnetwork. Recent studies suggested these three subnetworks display heterogeneous functional connectivity both in task state (Su et al. 2021) and resting state (Chen et al. 2017), while we did not find similar patterns in our dFNC results. However, we noticed that the differences in functional connectivity between T2DM and HC groups in the DMN were mainly reflected by differences in the PCUN. The PCUN is recognized as a key hub of the DMN, contributing to abstract cognitive processes, episodic memory retrieval, self-representation and conscious experience (Cavanna and Trimble 2006). Decreased connectivity of precuneus has been observed in mild cognitive impairment (MCI) (Mattioli et al. 2021), major depressive disorder (MDD) (Zhu et al. 2012) and early AD patients (Zhou et al. 2008).

The connectivity alterations were also detected in the TPN and SN. The TPN is an integrated region, including the executive control network (ECN), DAN and VAN (Boyatzis et al. 2014). The TPN, in contrast to the DMN, contains a series of areas that are activated during the goal-oriented task (Fox et al. 2005) and working memory task (Hampson et al. 2010), but deactivated in the resting state. The relationship between the TPN and DMN is generally regarded as anticorrelated, creating a fundamental neural constraint for cognitive function (Boyatzis et al. 2014). Hyper-activation in the TPN areas during tasks predicts worse accuracy (Grady et al. 2010) and TPN hyper-activation can be seen in the patients with mild cognitive impairment (Clement and Belleville 2010; Grady et al. 2003). The SN is a pivotal network for it can perceive and filter external stimuli and recruit relevant functional networks (Menon and Uddin 2010). It plays a crucial role in allocating attention, as well as switching between internally and externally directed cognition. Specifically, it can mediate the activation balance between the DMN and the ECN, depending on cognitive tasks or the resting state (Seeley, 2019; Chand et al. 2017). Although a growing number of studies on neurodegenerative diseases have focused on SN connectivity, the conclusions are often contradictory in different studies (Badhwar et al. 2017; Kim and Kim, 2021). For instance, some studies find increased SN connectivity in AD patients (Balthazar et al. 2014; Zhou et al. 2010), while other studies indicate decreased connectivity (Filippi et al. 2013). To date, few studies paid attention to the connectivity of the TPN and SN in T2DM patients. Almost all these T2DM studies just focus on alterations in static functional connectivity, and the conclusions from different studies are not entirely consistent. Some studies (Yang et al. 2016b) showed impaired inter-network and intra-network connectivity of the ECN, DAN and SN in T2DM patients, while other studies observed decreased connectivity of the DAN, VAN (Xia et al. 2015), and SN (Zhang et al. 2021). But another study showed increased connectivity in the SN (Cui et al. 2016) of T2DM patients. Unlike previous studies, our results indicated that the inter-network connectivity of the RECN, DAN and VAN, which all belonged to TPN, was increased in the T2DM group. Notably, our findings demonstrated a typically opposite tendency between the TPN and DMN, that is, enhanced inter-network connectivity of the TPN but decreased inter-network connectivity of the DMN in the T2DM group, corroborating the antagonistic relationship between the TPN and DMN. Furthermore, we also noticed increased connectivity between RECN and SN in T2DM group. Hence, we speculate that dFNC analysis approach can capture more information that is meaningful for investigating the neural mechanism of T2DM.

### Validation analyses

Results obtained under different time window length and number of clusters showed good similarity to those in our main study. By summarizing the results of validation analyses under different parameters, we found that compared to the HC group, the T2DM group always occupied lower proportion, shorter FT and MDT in the high segregated connected state, but higher proportion, longer FT and MDT in the fully connected state. Moreover, FT and MDT in the high segregated connected state were positively correlated with MoCA scores, while that in the fully connected state were negatively correlated with MoCA scores. These also consistent with our main research conducted under a 44 s (88TRs) time window length and 4 clusters, indicating the high segregated connected state and fully connected state may the crux differences between T2DM and HC, which might be related to cognitive impairment. The inter-network and intra-network connectivity of DMN decreased, while those networks belonging to the TPN showed increased inter-network and intra-network connectivity in T2DM group were also confirmed in the validation analyses. The above results illustrated the reliability and repeatability of our dFNC analysis for T2DM and HC individuals.

### Limitations

Our research has several limitations. Firstly, the generalizability of our findings might be constrained due to the relatively small sample size of our investigation. To validate our observations, we may enlarge the sample size in subsequent research (by using, for instance, open databases). Additionally, we did not evaluate T2DM patients’ treatment regimens in this study since they are complicated and constantly changing. Future studies can take the therapeutic interventions into account to assess their impacts on the functional connectivity of T2DM patients. Finally, our study is a cross-sectional study, we are considering longitudinal studies in the future to monitor the functional connectivity alterations before and after treatment, it will be of great significance to establish the pivotal point of cognitive function impairment in T2DM patients and assess the clinical treatment effects, as well as to probe sensitive imaging indicators for T2DM cognitive deficit.

## Conclusion

In this study, we explored potential differences between T2DM and HC individuals using dynamic FNC analysis. We found that T2DM individuals occupied lower proportion and shorter FT/ MDT in a high segregated connected state, while longer FT/ MDT and significant negative correlations with MoCA scores in a fully connected state. In addition, our study revealed functional connectivity alterations in three crucial cognition-related networks including the DMN, TPN and SN in T2DM patients. We uncovered the opposite connectivity patterns of the DMN and TPN. We also determined the role of the PCUN as a core region of the DMN in altered functional network connectivity in T2DM. These findings confirmed that dFNC analysis can capture additional information about brain network connectivity alterations of T2DM patients, providing novel insights into the underlying mechanisms of neurocognitive impairment related to T2DM.

## Data Availability

The original imaging data used and/or analyzed during the current study will be made available from the corresponding authors based on a formal data sharing agreement and a research proposal.
